# Age and Vasodilator Response to Different Hyperemic Agents: Adenosine versus Contrast Medium

**DOI:** 10.31083/j.rcm2507239

**Published:** 2024-07-02

**Authors:** Domenico Galante, Stefano Migliaro, Federico Di Giusto, Gianluca Anastasia, Edoardo Petrolati, Andrea Vicerè, Giuseppe Zimbardo, Pio Cialdella, Enrico Romagnoli, Cristina Aurigemma, Francesco Burzotta, Carlo Trani, Roberto Martin-Reyes, Sergio Bravo Baptista, Daniel Faria, Nicolas Amabile, Luis Raposo, Filippo Crea, Antonio Maria Leone

**Affiliations:** ^1^Diagnostic, Interventional and Acute Cardiac Care Unit, Ospedale Isola Tiberina – Gemelli Isola, 00186 Rome, Italy; ^2^Clinical, Interventional and Hemodynamic Cardiology Unit, Aurelia Hospital, 00165 Rome, Italy; ^3^Cardiology Unit, C. and G. Mazzoni Hospital, 63100 AST Ascoli Piceno, Italy; ^4^Department of Internal Medicine, University of Genoa, 16132 Genova, Italy; ^5^Department of Cardiovascular and Pneumological Sciences, Università Cattolica del Sacro Cuore, 00168 Rome, Italy; ^6^Cardiologia e Unità terapia intensiva, Policlinico Casilino, 00169 Rome, Italy; ^7^Department of Cardiovascular Sciences Policlinico Universitario Agostino Gemelli IRCCS, 00168 Rome, Italy; ^8^Unidad Integral de Cardiologia (UICAR). Hospital Universitario La Luz Quironsalud and Hospital Universitario Fundacion Jimenez Díaz Quironsalud, 28003 Madrid, Spain.; ^9^Cardiology Department, Hospital Prof. Doutor Fernando Fonseca, 2720-276 Amadora, Portugal; ^10^Centro Cardiovascular da Universidade de Lisboa, Faculdade de Medicina da Universidade de Lisboa, 1649-028 Lisboa, Portugal; ^11^Cardiology Department, L’Institut Mutualiste Montsouris, 75014 Paris, France; ^12^Department of Cardiology, Centro Hospitalar de Lisboa Ocidental, 1300-598 Lisbon, Portugal

**Keywords:** fractional flow reserve, contrast fractional flow reserve, vasodilatory response, age related impaired microcirculation

## Abstract

**Background:**

Age-related remodelling has the potential to affect the microvascular response to hyperemic stimuli. However, its precise effects on the vasodilatory response to adenosine and contrast medium, as well as its influence on fractional flow reserve (FFR) and contrast fractional flow reserve (cFFR), have not been previously investigated. We investigate the impact of age on these indices.

**Methods:**

We extrapolated data from the post-revascularization optimization and physiological evaluation of intermediate lesions using fractional flow reserve (PROPHET-FFR) and The Multi-center Evaluation of the Accuracy of the Contrast MEdium INduced Pd/Pa RaTiO in Predicting (MEMENTO) studies. Only lesions with a relevant vasodilatory response to adenosine and contrast medium were considered of interest. A total of 2080 patients, accounting for 2294 pressure recordings were available for analysis. The cohort was stratified into three age terciles. Age-dependent correlations with FFR, cFFR, distal pressure/aortic pressure (Pd/Pa) and instantaneous wave-free ratio (iFR) were calculated. The vasodilatory response was calculated in 1619 lesions (with both FFR and cFFR) as the difference between resting and hyperaemic pressure ratios and correlated with aging. The prevalence of FFR-cFFR discordance was assessed.

**Results:**

Age correlated positively to FFR (r = 0.062, *p* = 0.006), but not with cFFR (r = 0.024, *p* = 0.298), Pd/Pa (r = –0.015, *p* = 0.481) and iFR (r = –0.026, *p* = 0.648). The hyperemic response to adenosine (r = –0.102, *p* ≤ 0.0001) and to contrast medium (r = –0.076, *p* = 0.0023) showed a negative correlation with age. When adjusted for potential confounders, adenosine induced hyperaemia was negatively associated with age (*p* = 0.04 vs *p* = 0.08 for cFFR). Discordance decreased across age terciles (14.64% vs 12.72% vs 10.12%, *p* = 0.032).

**Conclusions:**

As compared to adenosine, contrast induced hyperaemia appeared to be less affected by age. cFFR may be considered a more stable and reproducible tool to assess epicardial stenosis in elderly patients.

**Clinical Trial Registration:**

PROPHET-FFR STUDY, Clinicaltrials.gov (NCT05056662).

## 1. Introduction

In the last decades, average life expectancy has increased, and consequently elderly patients represent an important proportion of those undergoing coronary angiography and eventually percutaneous coronary intervention. Functional evaluation using fractional flow reserve (FFR) or instantaneous wave-free ratio (iFR) is the gold standard for the assessment of intermediate coronary lesions [1]. Notably, these indexes will yield discordant results in about 15% of lesions [2, 3], arising questions about their accuracy in specific settings.

Aging is a well-known risk factor for microvascular dysfunction, secondary to a pro-inflammatory status and several other conditions, such as diabetes, hypertension and left ventricular hypertrophy, which are more prevalent in the elderly and affects vascular compensatory capacity [4, 5, 6].

Recent data confirmed the pivotal role of microcirculation in the vasodilatory response to hyperaemic agents [5, 6, 7, 8], and the influence of a pathological microvascular remodelling on FFR has been already addressed [4]. However, there is another validated hyperaemic index, contrast medium fractional flow reserve (cFFR) [9, 10] which relies on a milder hyperaemia than the one achieved by adenosine [7] and for which the relationship with aging and impaired microcirculation is unknown.

The purpose of our study was to investigate the impact of ageing on the vasodilatory response to different hyperaemic agents (adenosine vs contrast-medium). At the same time, we focused on the relationship between ageing and both hyperaemic (FFR, cFFR) and non- hyperaemic (distal pressure/aortic pressure (Pd/Pa), iFR) pressure-derived coronary physiology indices.

## 2. Methods

### 2.1 Study Design and Population

We analyzed pooled data from the post-revascularization optimization and physiological evaluation of intermediate lesions using fractional flow reserve (PROPHET-FFR) and The Multi-center Evaluation of the Accuracy of the Contrast MEdium INduced Pd/Pa RaTiO in Predicting (MEMENTO)-FFR studies. Details on the design, inclusion and exclusion criteria and results of these studies have been reported previously [11, 12]. In brief, the PROPHET-FFR study (Clinicaltrials.gov NCT05056662, ethical lot number, ID CE 3237) was a single centre, ambispective study of 1322 patients and 1591 lesions, evaluating the feasibility and the clinical efficacy of physiology-guided percutaneous coronary intervention (PCI) in patients with both chronic and stabilized acute coronary syndromes and at least one functionally tested intermediate coronary lesion, in which FFR, cFFR and several non-hyperaemic pressure ratio (NHPR) were evaluated. Concerning the acute coronary syndrome (ACS) patients, the functional evaluation was performed in non-culprit lesions during the same procedure in the case of non-ST segment elevation (NSTE)-ACS and in a deferred procedure, at least 72 h after admission, in the case of ST segment elevation (STE)-ACS.

The MEMENTO-FFR study was an international, multicenter, non-randomized, retrospective registry of 926 patients and 1026 coronary lesions evaluating the accuracy of cFFR in predicting FFR in patients with coronary artery disease in whom physiological lesion assessment was clinically indicated.

The studies were approved by the local ethics committees and conformed to the Declaration of Helsinki.

**Fig. 1.**
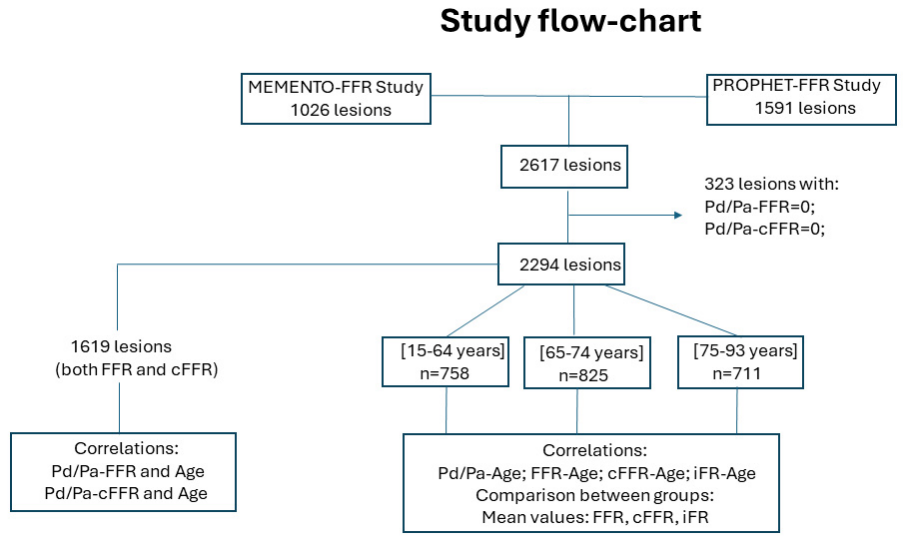
**Study flow-chart.** FFR, fractional flow reserve; cFFR, contrast fractional flow reserve; iFR, instantaneous wave-free ratio; Pd, distal pressure; Pa, aortic pressure; PROPHET-FFR, post-revascularization optimization and physiological evaluation of intermediate lesions using fractional flow reserve; MEMENTO-FFR, The Multi-center Evaluation of the Accuracy of the Contrast MEdium INduced Pd/Pa RaTiO in Predicting fractional flow reserve.

Lesions with absent vasodilator response to adenosine (Pd/Pa-FFR = 0) or contrast (Pd/Pa-cFFR = 0) were excluded from the analysis in order to minimize confoundment due to unmeasured variables related to induction of hyperaemia or measurement error (Fig. 1).

### 2.2 Functional Assessment 

#### 2.2.1 Hyperemic Index and Vasodilatory Response

When operators decided to assess the functional significance of an angiographically intermediate coronary stenosis, a dedicated guidewire (PressureWire™ Certus™ or Aeris™; St. Jude Medical/Abbott, St. Paul, MN, USA; PrimeWire™ or Verrata® wires; Volcano Corporation/Philips, Rancho Cordova, CA, USA) with a pressure sensor tip was advanced distally to the target lesion. Baseline pressures both distally to the lesion and at the coronary origin were measured to obtain basal Pd/Pa ratio. Subsequently, hyperemia was induced first by contrast medium injection, and second by adenosine infusion.

cFFR was calculated as the lowest ratio of distal coronary pressure divided by aortic pressure registered during the first 10 seconds after injection of a fixed dose of radiographic contrast medium (low osmolar non-ionic agent, 6 mL). Hemodynamic significance was defined as cFFR ≤0.83. After the acquisition of the valid cFFR value, a flushing of the guiding catheter with saline was performed in order to avoid pressure damping due to contrast medium viscosity and to restore resting conditions.

FFR was calculated after obtaining hyperemia with intravenous or intracoronary injection of adenosine. When adenosine was administered through the I.V. route, a standard 140 mcg/kg/min dose was used, whilst for the I.C. route incremental boli of adenosine from 60 mcg to 300 mcg (up to a maximum of 600 mcg) were administered, as tolerated. A lesion was defined as functionally significant if FFR was ≤0.80.

Every pressure tracing was thoroughly reviewed for possible technical issues and guidewire drift was checked after each measurement (acceptable drift +/– 0.02). Any measurement that did not meet these standards was repeated. Quantification of vasodilator response to different hyperemic agents (adenosine and contrast-medium) was calculated as the difference between baseline Pd/Pa and FFR and cFFR values respectively.

#### 2.2.2 Non Hyperemic Index

iFR was automatically calculated by the Volcano Software, as the Pd/Pa ratio in the time period between 25% into diastole and 5 ms before diastole ending [13]. A lesion was defined as functionally significant if iFR was ≤0.89.

### 2.3 Age Strata and Pressure Derived Indices

Patients were stratified into three groups based on age terciles. All correlations between intracoronary physiology indices and age were addressed. The mean values of FFR, cFFR, Pd/Pa and iFR were evaluated and compared between age groups.

In order to avoid the possible confounding effect of the case mix, the vasodilatory response to adenosine and contrast medium was evaluated only in lesions for which both FFR and cFFR values were available and subsequently correlated with aging (Fig. 1).

The FFR/cFFR discordance, defined as “positive” or “negative” in the presence of lesions with FFR ≤0.8 + cFFR ≥0.83 and FFR >0.80 + 0.83 <cFFR respectively, was estimated and compared between groups.

### 2.4 Statistical Analysis

Categorical variables were reported as counts and percentages while continuous variables were reported as means and standard deviation or median with interquartile range according to normality. Normality was checked using the Kolmogorov Smirnov test. Differences among categorical variables were assessed using Pearson χ^2^ test. In contrast, differences among continuous variables among the three groups were calculated using the independent analysis of variance (ANOVA) or Kruskal Wallis test according to normality. Correlations between coronary physiology indices and age were assessed by the Pearson correlation coefficient and subsequently adjusted for several potential confounders in a multivariate linear regression including angiographical severity of the interrogated lesions (in percentage), interrogated lesion in the left anterior descending (LAD) artery, diabetes, previous myocardial infarction (MI), multivessel disease, chronic kidney disease (CKD), left ventricular dysfunction (defined as an ejection fraction below 40%) and clinical presentation as an ACS. The hyperaemic response to adenosine and contrast medium was correlated with age and confirmed in a multivariate linear regression including the same confounders described previously. Logistic regression analysis was applied to relate a broad range of admission parameters to predict FFR-cFFR discordance. Statistical analysis was performed using commercially available software (Stata 13.2 [StataCorp, College Station, TX, USA]). Statistical significance was defined as a two-sided *p*-value < 0.05.

## 3. Results

### 3.1 Population Characteristics

A total of 2080 patients were evaluated. The clinical characteristics of the patients are shown in Table 1. Stratification into age terciles resulted in the following groups–1st tercile: 15–64 years (n = 696); 2nd tercile: 65–74 years (n = 749); and 3rd tercile: 75–93 years (n = 635).

**Table 1.** **General characteristics of the study population**.

		Patients (n = 2080)	[15–64] years (n = 696)	[65–74] years (n = 749)	[75–93] years (n = 635)	*p*-value
Baseline demographics						
	Age, years	68.36 ± 10.24	56.83 ± 6.57	69.60 ± 2.95	79.53 ± 3.63	-
	Male (%)	72.98	79.45	69.69	69.76	<0.001*
Clinical characteristics						
	Hypertension (%)	80.85	71.37	84.70	86.73	<0.001*
	Diabetes mellitus (%)	30.16	25.94	32.88	31.60	0.010*
	Dyslipidemia (%)	62.84	58.38	68.01	61.65	<0.001*
	CKD (%)	8.16	4.76	8.43	10.72	0.017*
	EF <40% (%)	4.49	2.86	3.47	6.97	0.016*
	Aspirin (%)	87.18	85.71	88.64	86.96	0.359
	Clopidogrel (%)	51.65	50.41	53.15	51.14	0.648
	Beta-blockers (%)	67.17	68.24	68.12	64.93	0.440
	ARBs (%)	68.61	66.94	72.38	65.84	0.047*
	Insulin (%)	6.38	5.94	5.53	7.74	0.332
	Oral Antidiabetic drugs (%)	18.92	15.68	20.76	19.78	0.118
	Previous PCI (%)	43.14	40.81	45.02	44.09	0.500
	Previous MI (%)	25.01	25.38	24.62	25.08	0.947
	ACS (%)	30.38	31.75	27.77	31.97	0.150
	Multivessel disease (%)	27.92	30.15	27.77	25.74	0.279

CKD, chronic kidney dysfunction; EF, ejection fraction; ARBs, angiotensin receptor blockers; PCI, percutaneous coronary intervention; MI, myocardial infarction; ACS, acute coronary syndromes; * means *p*-value < 0.05.

More than half of the population was male (73%) and the main clinical presentation was chronic coronary syndrome (69.6%). As expected, most of the traditional coronary artery disease (CAD) risk factors were more prevalent in the older age group, while male sex was more prevalent in the younger age tertile (Table 1).

### 3.2 Lesions Characteristics

A total of 2294 lesions were evaluated. Characteristics of the lesions are shown in Table 2.

**Table 2.** **General characteristics of the epicardial stenosis and pressure-derived indices of functional stenosis relevance**.

		Lesions (n = 2294)	[15–64] years (n = 758)	[65–74] years (n = 825)	[75–93] years (n = 711)	*p*-value
Left anterior descending (LAD)		60.56	60.51	59.66	61.63	0.734
Left circumflex (LCx)		14.30	13.25	16.21	13.22	0.154
Right coronary artery (RCA)		25.14	26.24	24.13	25.14	0.633
Stenosis severity %		55.67 ± 10.56	55.80 ± 10.56	55.70 ± 10.74	55.51 ± 10.38	0.871
Physiological indices						
	Pd/Pa	0.93 ± 0.05	0.93 ± 0.04	0.93 ± 0.05	0.93 ± 0.05	0.474
	iFR	0.90 ± 0.07	0.90 ± 0.06	0.91 ± 0.07	0.89 ± 0.08	0.170
	cFFR	0.87 ± 0.07	0.87 ± 0.07	0.87 ± 0.08	0.87 ± 0.07	0.753
	FFR	0.84 ± 0.08	0.84 ± 0.08	0.84 ± 0.08	0.85 ± 0.07	0.132

Pd, distal pressure; Pa, aortic pressure; FFR, fractional flow reserve; iFR, instantaneous wave-free ratio; cFFR, contrast fractional flow reserve; Pd/Pa, resting distal to aortic pressure ratio.

Overall, the LAD artery was the most frequent lesion location accounting for 61% (n = 1399) of cases, followed by the right coronary artery (RCA) and left circumflex artery (LCx) at 25% (n = 573) and 14% (n = 322) respectively. Mean angiographic stenosis was 56 ± 11% and there was no significant intergroup difference regarding the interrogated coronary artery and severity of the lesions (Table 2). Coronary stenoses were of intermediate severity, both angiographically (mean diameter stenosis 56 ± 11%) and physiologically (mean Pd/Pa 0.93 ± 0.05, iFR 0.90 ± 0.07, cFFR 0.87 ± 0.07 and FFR 0.84 ± 0.08) (Table 2).

### 3.3 Effect of Age on Pressure-Based Functional Indices

We found a weak, albeit significant, positive correlation between age and FFR (r = 0.062, 95% CI: 0.018 to 0.106, *p* = 0.006) (Fig. 2A), while neither cFFR (r = 0.024, 95% CI: – 0.021 to 0.068, *p* = 0.2979) (Fig. 2B), Pd/Pa (r = –0.015, 95% CI: –0.056 to 0.026, *p* = 0.4812) (Fig. 2C) nor iFR (r = –0.026, 95% CI: –0.138 to 0.086, *p* = 0.6486) (Fig. 2D) showed any correlation with age. The impact of aging on FFR was independent of ACS, presence of diabetes mellitus, multivessel disease, previous myocardial infarction, CKD, left ventricular dysfunction, angiographic lesion severity and LAD location (Table 3). In a multivariate regression model, LAD location and % stenosis of the lesion were negatively correlated with all functional indices (Table 3).

**Fig. 2A.**
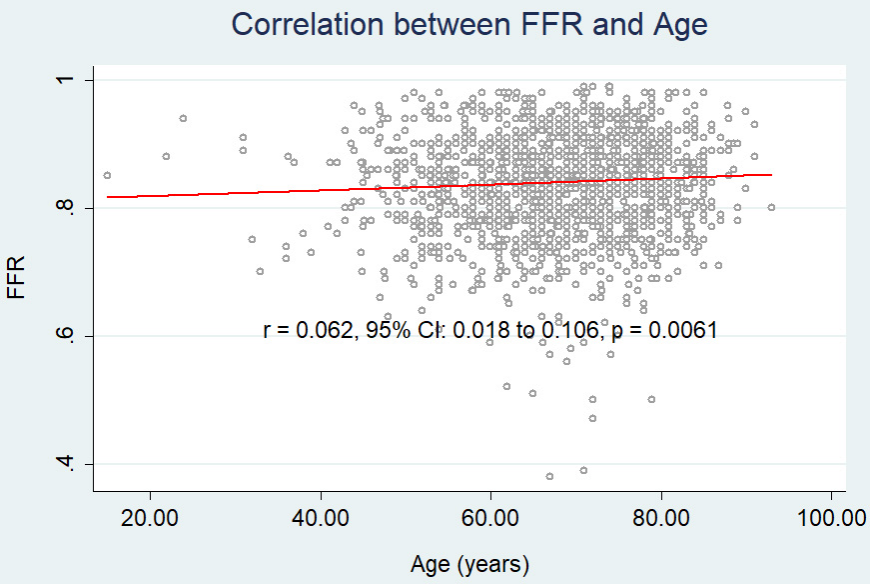
**Correlation between age and FFR.** FFR, fractional flow reserve.

**Fig. 2B.**
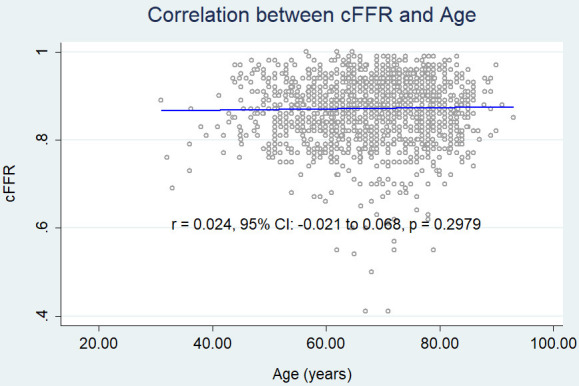
**Correlation between age and cFFR.** cFFR, contrast fractional flow reserve.

**Fig. 2C.**
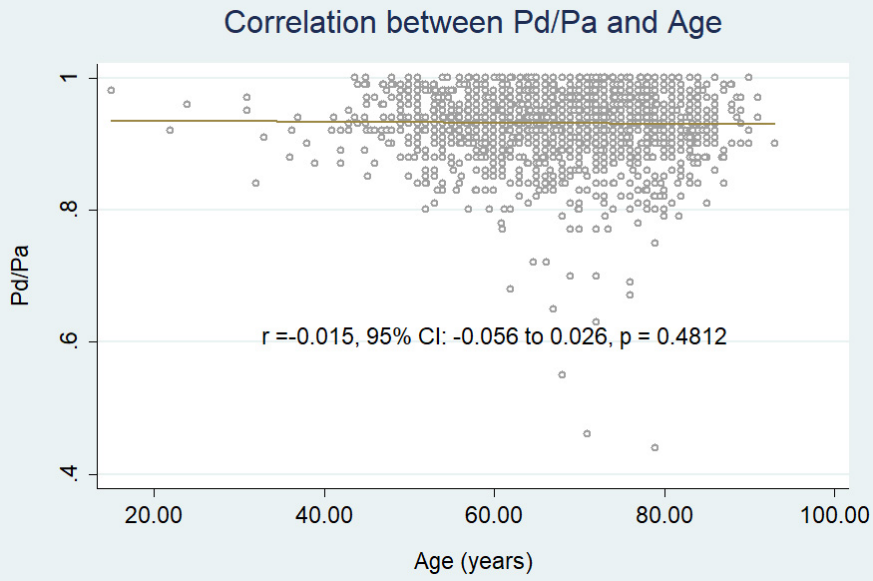
**Correlation between age and Pd/Pa.** Pd, distal pressure; Pa, aortic pressure.

**Fig. 2D.**
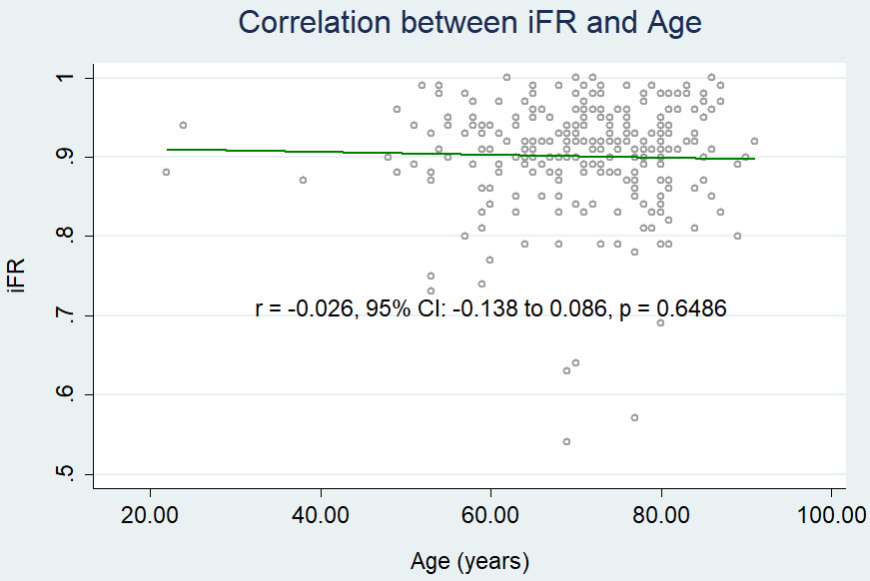
**Correlation between age and iFR.** iFR, instantaneous wave-free ratio.

**Table 3.** **Multiple linear regression for predictors of FFR, cFFR, iFR, Pd/Pa**.

	Linear regression FFR		Linear regression cFFR		Linear regression iFR		Linear regression Pd/Pa	
	Beta coefficient	*p* value	Beta coefficient	*p* value	Beta coefficient	*p* value	Beta coefficient	*p* value
Age	0.0004	0.019*	0.0002	0.171	0.0002	0.569	–0.0001	0.674
Diabetes mellitus	–0.0053	0.190	–0.0068	0.083	–0.0102	0.248	–0.0034	0.208
Previous MI	–0.0005	0.904	–0.0050	0.263	–0.0091	0.369	–0.0021	0.460
Stenosis (%)	–0.0025	<0.001*	–0.0022	<0.001*	–0.0018	<0.001*	–0.0015	<0.001*
ACS	–0.0051	0.212	0.0016	0.688	–0.0114	0.227	–0.0001	0.967
EF <40%	–0.0072	0.437	–0.0269	0.004*	–0.0184	0.339	–0.0049	0.402
CKD	–0.0026	0.725	–0.0144	0.025*	–0.0022	0.873	–0.0144	0.001*
Multivessel Disease	–0.0284	<0.001*	–0.0228	<0.001*	–0.0186	0.077	–0.0786	0.008*
LAD	–0.0446	<0.001*	–0.0441	<0.001*	–0.0591	<0.001*	–0.0360	<0.001*

FFR, fractional flow reserve; iFR, instantaneous wave-free ratio; cFFR, contrast fractional flow reserve; Pd/Pa, resting distal to aortic pressure ratio; MI, myocardial infarction; ACS, acute coronary syndromes; CKD, chronic kidney dysfunction; EF, ejection fraction; LAD, left anterior descending; * means *p*-value < 0.05.

**Fig. 3.**
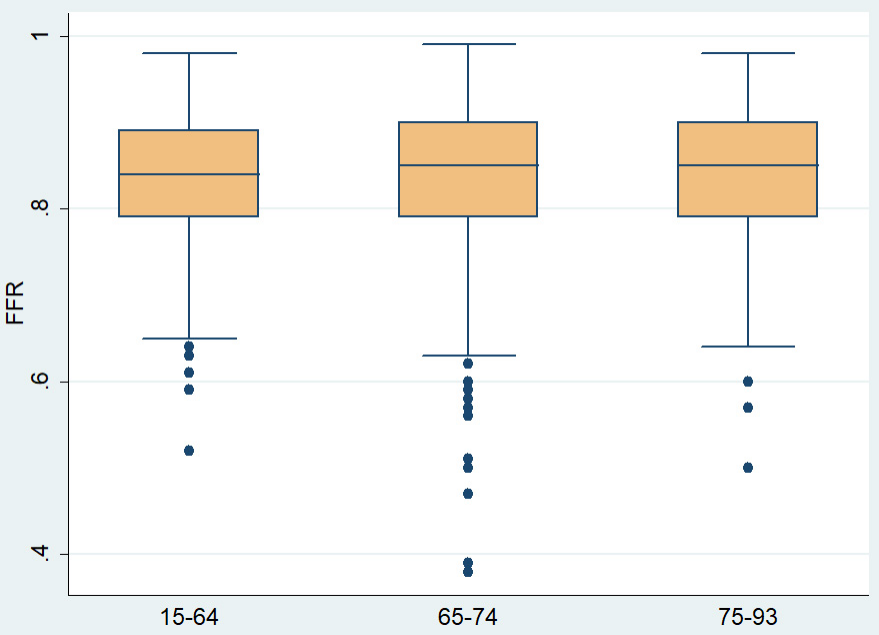
**Mean FFR values across different age terciles.** FFR, fractional flow reserve.

FFR was significantly higher in the 3rd age tercile as compared to the 1st tercile (0.85 ± 0.07 vs 0.84 ± 0.08 *p* = 0.034) (Fig. 3, **Supplementary Table 1**). This was not the case for cFFR, Pd/Pa and iFR which did not differ between age terciles (Table 2).

### 3.4 Ageing and Hyperemic Responses to Hyperemic Agents

**Fig. 4.**
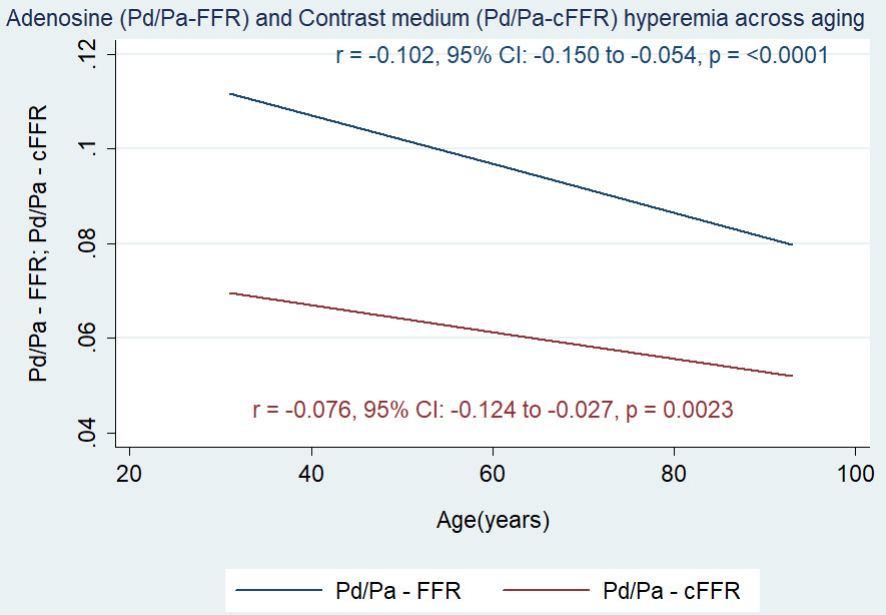
**Correlation between age and both adenosine-induced (Pd/Pa-FFR) and contrast medium-induced (Pd/Pa-cFFR) hyperemia.** FFR, fractional flow reserve; cFFR, contrast fractional flow reserve; Pd, distal pressure; Pa, aortic pressure.

The vasodilatory response was estimated in 1619 lesions (70.6%). While there was no significant age-dependent correlation between Pd/Pa and age (r = –0.023, 95% CI: –0.072 to 0.025, *p* = 0.3465), we observed a significant and negative correlation between age and both hyperemic response to adenosine (r = –0.102, 95% CI: –0.150 to –0.054, *p* ≤ 0.0001) and contrast medium (r = –0.076, 95% CI: –0.124 to –0.027, *p* = 0.0023) (Fig. 4). However, when considered in a multivariate analysis including angiographic lesion severity, acute clinical presentations, lesion location on LAD artery and other possible confounder factors, only adenosine-induced hyperemia, as assessed by resting Pd/Pa-FFR difference, remained negatively correlated with age (Table 4).

**Table 4.** **Multiple linear regression for predictors of Pd/Pa-FFR and Pd/Pa-cFFR**.

	Linear regression Pd/Pa-FFR		Linear regression Pd/Pa-cFFR	
	Beta coefficient	*p* value	Beta coefficient	*p* value
Age	–0.0004	0.044*	–0.0002	0.077
Diabetes mellitus	0.0050	0.181	0.0038	0.169
Previous MI	–0.0004	0.916	–0.0005	0.855
Stenosis (%)	0.0010	<0.001*	0.0006	<0.001*
ACS	0.0028	0.73	0.0007	0.805
EF <40%	0.0186	0.04*	0.0187	0.005*
CKD	–0.0024	0.727	–0.0053	0.297
Multivessel Disease	0.0209	<0.001*	0.0086	0.007*
LAD	0.0089	0.023*	0.0079	0.005*

FFR, fractional flow reserve; cFFR, contrast fractional flow reserve; Pd/Pa, resting distal to aortic pressure ratio; MI, myocardial infarction; ACS, acute coronary syndromes; EF, ejection fraction; CKD, chronic kidney dysfunction; LAD, left anterior descending; * means *p*-value < 0.05.

### 3.5 Discordance

**Fig. 5.**
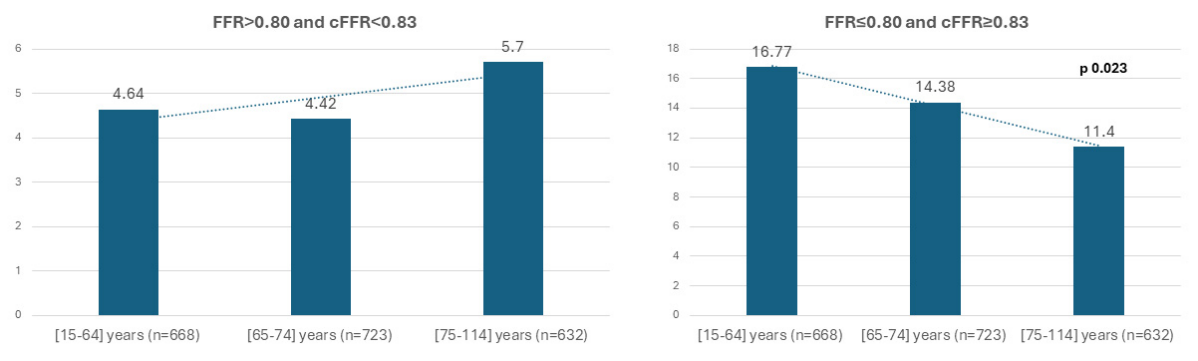
**Positive (FFR ≤0.80 and cFFR ≥0.83) and negative (FFR >0.80 and 0.83 <cFFR) FFR/cFFR discordance across the age terciles.** FFR, fractional flow reserve; cFFR, contrast fractional flow Reserve.

FFR and cFFR discordance was defined as “negative” in the presence of lesions with FFR >0.80 + cFFR <0.83 and “positive” with FFR ≤0.8 + cFFR ≥0.83, respectively. While the prevalence of negative discordance was not statistically different among different terciles (4.49% 1st tercile, 4.00% 2nd tercile, 5.34% 3rd tercile, *p* = 0.449), the prevalence of positive discordance showed a significant decline with increasing age (14.64% 1st tercile vs 12.72% 2nd tercile vs 10.12% 3rd tercile, *p* = 0.032) (Fig. 5, **Supplementary Table 2**). Age was confirmed as a predictor of “positive” discordance in a logistic regression model including a board of potential clinical confounders (**Supplementary Table 3**).

## 4. Discussion

Several technical, clinical, angiographic and hemodynamic factors may influence the output of functional assessment of epicardial stenosis [4, 5, 6]. The presence of microvascular dysfunction represents an important confounder during epicardial physiological assessment [8]. In the present study, we focused on the influence of age on the vasodilatory response to different hyperaemic agents (adenosine versus contrast medium). We have simultaneously analyzed the impact of age on the values of both hyperaemic (FFR and cFFR) and non-hyperaemic indices (Pd/Pa, iFR).

The main results are the following:

-Age appeared to be associated with a decreased hyperaemic response to adenosine, which translates into a higher FFR value;

-Non-hyperemic pressure derived ratios (Pd/Pa, iFR) are confirmed not to be significantly affected by age;

-Contrast medium hyperemia is less affected by age and consequently, cFFR values do not change significantly across the age strata.

We observed an age-dependent reduction in vasodilatory response to adenosine, and we hypothesize that it may be due to an impairment of the vasodilatory capacity of the coronary microcirculation. Aging is a well-known risk factor for microvascular dysfunction, as it induces functional and structural remodelling of all components of the microvascular domain [7, 14].

Age-induced inflammatory state is responsible for endothelial dysfunction and promotes the recruitment of inflammatory cells and cytokine release. As such, older patients experience pathological derangement of vascular and perivascular environment that ultimately leads to impairment of local regulation of microvascular perfusion (by impairing endothelium-mediated, flow-induced arteriolar vasodilation, and angiogenesis) [15, 16]. Moreover, there is a negative impact on oxygen delivery, cellular bioenergetics and scavenging capacity that results in microvascular rarefaction [15, 16, 17, 18]. The direct consequence of these changes is the increase in minimal microvascular resistance under maximal hyperaemia that translates into a partial and progressive loss of coronary reserve [7, 14, 15, 16, 17, 18].

Our results are in line with previous studies reporting a negative correlation between age and coronary flow reserve (CFR), evaluated by thermodilution technique [7, 14].

We identified a modest yet positive correlation between FFR and age, suggesting a potential impact of aging on adenosine-related indices. This connection was reinforced by the observation of higher FFR values in older patients, alongside a notable decline in hyperemic response to adenosine across age groups. Even after adjusting for other significant factors like LAD location and stenosis severity through multivariate regression analysis, advancing age remained linked to both FFR and adenosine-related hyperemia; this was not the case for cFFR and non-hyperemic indices. These diverse strands of evidence affirm this association, addressing initial reservations regarding the perceived weak correlation between age and FFR. The present findings align seamlessly with recent literature on the topic. A study by Faria *et al*. [13] demonstrated a similar correlation between age and FFR (r = 0.08, *p* = 0.015) in a small cohort of patients (n = 598).

To the best of our knowledge, this is the first study to show a preserved age-related vasodilatory response to contrast medium injection, with cFFR values being comparable across the age terciles. cFFR is a relatively novel hyperaemic index which uses a contrast medium to induce hyperaemia relying on its osmolality [9, 10]. Contrast-induced hyperaemia has been considered “submaximal” because it is significantly less intense than that induced by adenosine [9, 10], on average conditions cFFR accuracy for disclosing significant lesions was proven in the RINASCI [19] study while other studies documented its superiority to resting Pd/Pa and iFR in predicting FFR [20, 21, 22, 23].

A possible explanation of this different behaviour of adenosine and contrast medium’s hyperaemia across the age spectrum could be related to the physical properties of these agents and also different mechanisms of action, potentially less influenced by intrinsic microvascular dysfunction. While adenosine-induced hyperaemia is principally driven by non-endothelium mediated mechanisms (A2 receptors of smooth muscular cells) dampened by the presence of fibrosis and capillary rarefaction, contrast medium induces hyperaemia by its osmolarity causing the opening of K(ATP) channels in the vascular endothelium and for which the influence of microvascular remodelling is not clear [9, 10].

Another conceivable explanation could be related to the desensitization phenomena. Previous studies revealed a reduction of adenosine’s effect in elderly patients [24, 25]. Experimental models have shown greater local release of adenosine in the coronary microcirculation of older animals, leading to a reduction of expression of adenosine receptors and limiting the effectiveness of an exogenous administration [26]. This was not documented for contrast medium.

Furthermore, the presence of age-related diseases such as diabetes and hypertension, could contribute to microvascular dysfunction, particularly affecting adenosine-induced hyperemia. Histopathological examinations in diabetic animals have unveiled various coronary vessel abnormalities, including arteriolar thickening, perivascular connective tissue accumulation, capillary microaneurysms, and reduced capillary density [27]. These findings culminate in impaired coronary vasodilation upon administration of exogenous adenosine, suggesting a potential connection between compromised metabolic vasodilation and diabetes mellitus [27]. Similarly, studies on left ventricular hypertrophy have shown an inverse correlation between capillary density and hypertrophy severity, associated with impaired adenosine-induced flow augmentation [4, 5, 6].

Differences in the sensitivity to adenosine can provide further clues into the discordance phenomenon. Recent studies showed that discordance between FFR and cFFR is observed in about 10–15% of patients [2]. In the rare case of discordance between FFR and cFFR, the former is reliable and more accurately predict worse outcomes [26]. Specifically, our group has previously demonstrated that FFR+/cFFR- patients showed a prognosis similar to FFR-/cFFR- patients while FFR-/cFFR+ patients showed a prognosis similar to FFR+/cFFR+ patients [28].

In the present study, we described a significant reduction of positive discordance (defined as FFR ≤0.8 and cFFR ≥0.83) and increasing age suggesting that aging may be one of the most important factors associated with FFR-cFFR discrepancy. Conversely, non-hyperaemic indices, such as Pd/Pa and iFR, appeared to be unrelated to aging, in line with the recently reported observations [13].

Our findings suggest for the first time a similar behaviour between hyperaemic (cFFR) and non-hyperaemic indices (iFR, Pd/Pa) in patients with possible impairment in microcirculation, such as older aged groups.

Overall, in this particular setting, cFFR could be considered to incorporate the best characteristic of the most important hyperemic (FFR) and non-hyperemic (iFR, Pd/Pa) indices, in that it still allows some assessment of residual vasodilatory capacity without compromising accuracy.

## 5. Limitations

The present analysis was based on retrospective data from PROPHET-FFR and MEMENTO studies and our conclusions are mainly hypothesis-generating rather than hypothesis testing. However, there was a clear biological and clinical rationale for performing this analysis, as the microcirculatory modifications associated with age had been previously described. Another limitation might be the lack of an invasive assessment of the microvascular resistance, which would have provided a more comprehensive insight into this condition.

## 6. Conclusions 

Aging seems to be associated with a decreased vasodilatory response of the microcirculation to adenosine administration. This was not observed for contrast medium-induced hyperaemia. FFR showed a weak and positive correlation with age probably leading to elevated FFR values while cFFR didn’t change across the age spectrum. This fact may influence the degree of concordance between these two hyperaemic indices in terms of functional stenosis classification. Between hyperaemic indexes, cFFR is less affected by age.

## 7. Impact on Daily Practice

Elderly patients present higher FFR values due to an impaired response to adenosine. However, cFFR values and hyperaemic response to contrast-medium are not influenced by patient age. cFFR may be considered a more reliable and reproducible index than FFR in the evaluation of epicardial stenosis in this setting.

## Data Availability

The datasets used and/or analyzed during the current study are available from the corresponding author on reasonable request.

## Abbreviations

CAD, coronary artery disease; ACS, acute coronary syndrome; CCS, chronic coronary syndromes; NSTE- ACS, non-ST segment elevation acute coronary syndromes; STE-ACS, ST segment elevation acute coronary syndromes; EF, ejection fraction; CKD, chronic kidney disease; MI, myocardial infarction; PCI, percutaneous coronary intervention; FFR, fractional flow reserve; cFFR, contrast fractional flow reserve; iFR, instantaneous wave-free ratio; LAD, left anterior descending; RCA, right coronary artery; LCx, left circumflex; Pa, aortic pressure; Pd, distal pressure.

## Supplementary Material

Supplementary material associated with this article can be found, in the online version, at https://doi.org/10.31083/j.rcm2507239.
