# Supplementary material for: Age and Vasodilator Response to Different Hyperemic Agents: Adenosine versus Contrast Medium
Source: Rev Cardiovasc Med. 2024 Jul 2;25(7):239. doi: 10.31083/j.rcm2507239 (PMC11317353; doi:10.31083/j.rcm2507239)
Supplement: Supplementary file 1 [file 2153-8174-25-7-239-s1.docx]

**Supplementary Table 1. Physiological characteristics of the epicardial stenosis with FFR evaluation.**

|  | Lesions (n=2294) | | [15-64] years (n=758) | | [65-74] years (n=825) | | [75-93] years (n=711) | | *p*-value | |
| --- | --- | --- | --- | --- | --- | --- | --- | --- | --- | --- |
| FFR value | 0.83±0.08 | 0.84±0.08 | | 0.84±0.08 | | 0.85± 0.07 | | 0.132 | |  |
| - |  | 0.84±0.08 | | 0.84±0.08 | |  | | 0.337 | |  |
| - |  | 0.84±0.08 | |  | | 0.85± 0.07 | | 0.034* | |  |
|  |  |  | | 0.84±0.08 | | 0.85± 0.07 | | 0.281 | |  |

**FFR: fractional flow reserve**

**Supplementary Table 2. Prevalence of FFR/cFFR discordance between age terciles.**

|  | Lesions (n=2294) | [15-64]  years (n=758) | [65-74]  years (n=825) | [75-93]  years (n=711) | *p*-value |
| --- | --- | --- | --- | --- | --- |
| Discordance | | | | | |
| FFR>0.80 and cFFR<0.83 | 4.58 | 4.49 | 4.00 | 5.34 | 0.449 |
| FFR≤0.80 and cFFR≥0.83 | 12.55 | 14.64 | 12.73 | 10.13 | 0.032* |

**Supplementary Table 3. Logistic regression for predictors of FFR and cFFR discordance.**

|  | FFR>0.80 and cFFR<0.83 | | | | FFR≤0.80 and cFFR≥0.83 | | | |
| --- | --- | --- | --- | --- | --- | --- | --- | --- |
|  | Coeff. | SD | p | 95% IC | Coeff. | SD | p | 95% IC |
| Age | 0.007 | 0.013 | 0.601 | -0.018±0.032 | -0.027 | 0.008 | 0.002* | -0.04±0.01 |
| ACS | -0.434 | 0.3 | 0.148 | -1.022±0.154 | 0.25 | 0.196 | 0.201 | -0.133±0.634 |
| Diabetes Mellitus | -0.112 | 0.266 | 0.674 | -0.634±0.410 | -0.196 | 0.198 | 0.322 | -0.584±0.192 |
| % stenoses | 0.054 | 0.012 | <0.01* | 0.030±0.077 | 0.06 | 0.008 | <0.01* | 0.042±0.077 |
| LAD location | 0.233 | 0.298 | 0.432 | 0.349±0.817 | 1.24 | 0.267 | <0.01* | 0.717±1.767 |
| CKD | 1.144 | 0.316 | <0.01* | 0.522±1.759 | -0.2012 | 0.334 | 0.548 | 0.858±0.455 |
| Multiple lesions | 0.876 | 0.257 | <0.01* | 0.372±1.380 | 0.612 | 0.194 | <0.01* | 0.231±0.993 |
| EF<40% | 0.191 | 0.508 | 0.707 | -0.805±1.188 | 0.159 | 0.421 | 0.705 | -0.667±0.985 |
| Previous MI | 0.198 | 0.287 | 0.491 | 0.364±0.759 | -0.139 | 0.217 | 0.524 | 0.565±0.288 |

**FFR: fractional flow reserve, cFFR: contrast fractional flow reserve, MI: myocardial infarction, ACS: Acute coronary syndromes, EF: Ejection Fraction, CKD: Chronic Kidney dysfunction, LAD: left anterior descending.**
